# Supplementary material for: Simulating HIV transmission dynamics: An agent-based approach using NetLogo
Source: PLoS One. 2025 Sep 11;20(9):e0330456. doi: 10.1371/journal.pone.0330456 (PMC12425208; doi:10.1371/journal.pone.0330456)
Supplement: Attachment — Submitted filename: Response to Reviewers.pdf [file pone.0330456.s001.pdf]

José Antonio Ortega, Ph.D.
Academic Editor
PLOS ONE

July 5, 2025

Manuscript ID: PONE-D-24-55952
Manuscript Title: *Simulating HIV Transmission Dynamics: An Agent-Based Approach Using NetLogo*

Subject: *Response to Reviewers*.

Dear Dr. Ortega,

We sincerely thank you and the reviewer for the valuable feedback, which has strengthened our manuscript. Below we detail the actions taken to address the requested revisions:

1. We have named the files appropriately as:
    - *Response to Reviewers* (this document)
    - *Revised Manuscript With Track Changes*
    - *Manuscript* (clean version)
2. We followed the PLOS One LaTeX template in revising and formatting the manuscript.
3. We have included the required funder statement regarding Mindanao State University-Iligan Institute of Technology:
   "The funders had no role in study design, data collection and analysis, decision to publish, or preparation of the manuscript."
4. We confirm that the code and data are available at:
   https://medium.com/@tri.beleta/simulating-hiv-transmission-dynamics-an-agent-based-approach-using-netlogo-2eab9790e37e
5. Figure 1 is now clearly referred to in the text.
6. Tables 2, 3, and 5 are all explicitly referred to in the text to ensure clarity and consistency.
7. We corrected the authorship details in reference number 3 to fix the previous typographical error.

8. We complied with the addition of confidence intervals as requested. However, implementing a split-sample validation was challenging because the model uses constant parameter values throughout the simulated timeframe, meaning there is insufficient variation to meaningfully validate on separate samples.

9. All other minor revisions have been incorporated in sections 1 and 2. In addition, sections 1 and 2 have been edited to address grammatical and clarity concerns. Table 2 and Figures 4, 14, and 17 are now fully defined and accompanied by detailed captions for clarity.

We appreciate the opportunity to revise and resubmit, and we hope the manuscript now meets the journal's requirements.

Respectfully,

Orven E. Llantos, PhD
on behalf of all co-authors
